# Effectiveness comparisons of different Chinese herbal injection therapies for acute cerebral infarction: A protocol for systematic review and network meta-analysis

**DOI:** 10.1097/MD.0000000000021584

**Published:** 2020-08-07

**Authors:** Runmin Li, Ying Li, Bingchen Li, Haiyang Sun, Xinyu Liu, Xin Ge, Yuanxiang Liu, Jiguo Yang

**Affiliations:** a College of Traditional Chinese Medicine, Shandong University of Traditional Chinese Medicine, Jinan; b Nanchang University, Jiangxi; c Department of Neurology, Affiliated Hospital of Shandong University of Traditional Chinese Medicine; d College of First Clinical Medicine, Shandong University of Traditional Chinese Medicine; e College of Acupuncture and Massage, Shandong University of Traditional Chinese Medicine, Jinan, People's Republic of China.

**Keywords:** acute cerebral infarction, Chinese herbal injection, network meta-analysis, protocol

## Abstract

**Background:**

Acute cerebral infarction (ACI) has a high incidence, recurrence rate, and mortality. Chinese herbal injections (CHIs) are widely used in the substitution therapy of ACI. Due to the lack of randomized trials comparing the efficacy of various injections directly, it is still difficult to judge the relative efficacy. Therefore, we intend to conduct a network meta-analysis to evaluate the benefit among these CHIs.

**Methods:**

According to the retrieval strategies, randomized controlled trials (RCTs) on CHI therapies for ACI will be obtained from China National Knowledge Infrastructure, WanFang, Chinese Scientific Journals Database, PubMed, Embase and Cochrane Library, regardless of publication date or language. Studies were screened based on inclusion and exclusion criteria, and the Cochrane risk bias assessment tool will be used to evaluate the quality of the literature. The network meta-analysis will be performed in Markov Chain Monte Carlo method and carried out with Stata 14 and WinBUGS 1.4.3 software. Ultimately, the evidentiary grade for the results will be evaluated.

**Results:**

This study will compare the efficacy and safety of CHIs in the treatment of ACI, and give a more reasonable choice.

**Conclusion:**

Our findings will provide references for future clinical decision and guidance developing.

INPLASY registration number: INPLASY202060087.

## 1 Introduction

Acute cerebral infarction (ACI), also called acute ischemic stroke, refers to the syndrome of local cerebral ischemia, hypoxia, and neurological impairment occurring within 14 days caused by cerebrovascular disease.^[1–3]^ ACI has a high incidence, recurrence rate, and mortality; it accounts for 69.6% to 70.8% of stroke cases in China,^[4,5]^ with a prevalence rate of 2.19% and recurrence rate of 14.7%, and an annual death toll of approximately 1.96 per million.^[6]^ It is a public health problem that needs to be solved urgently.

Based on available evidence, rt-PA intravenous thrombolysis within 4.5 hours of symptom onset has clear benefits and is the preferred treatment option. However, due to the strict time window, less than 3% of patients benefit from the treatment.^[7]^ Reasonable application of antiplatelet, anticoagulation, and nutritional nerve treatment regimens can improve symptoms and reduce the recurrence rate but may increase the possibility of intracranial hemorrhage.^[8–10]^ To remedy these defects, traditional Chinese medicine (TCM) substitution therapy has been widely considered in China.

TCM hold that ACI is caused by blood stasis; thus, the use of blood circulation and blood stasis herbs has been given importance in the treatment of this condition.^[3]^ Small molecules refined from herbs are an effective component of Chinese herbal injections (CHIs). Modern pharmacological studies have demonstrated that this TCM dose form has a wide range of targets and high bioavailability, which are conducive to the timely treatment of ACI.^[11–13]^ In addition, a large number of studies have reported that CHIs can improve the neurological function and daily life activities of ACI patients.^[14–16]^ Microscopic mechanistic studies have suggested that CHIs can correct abnormal blood clotting systems and lipid metabolism by reducing homocysteine, C-reactive protein, D-dimer, fibrinogen, triacylglycerol, total cholesterol and low-density lipoprotein.^[17]^ In addition, studies have shown that CHIs can improve intracranial microcirculation by reducing the intima-media thickness and pulse wave velocity.^[18]^

Although randomized controlled trials (RCTs) of ACI treatments involving various CHIs have been frequently reported, due to the limitations of study design and scale, it is difficult to directly rank the efficacy of the drugs, so it is ignored in practical application. As a branch of traditional meta-analysis, network meta-analysis integrates existing studies to form an evidence network that can indirectly compare treatment benefits.^[19–21]^ In this study, network meta-analysis will be used to evaluate the efficacy and safety of CHIs, and the conclusion will further guide clinical practice strive for maximum benefit for patients.

## 2 Methods

### 2.1 Objectives and registration

This systematic review will aim to evaluate the effect and safety of CHI therapy for ACI. Our protocol has been registered on the International Platform of Registered Systematic Review and Meta-Analysis Protocols (INPLASY). The registration number was INPLASY202060087 (DOI: 10.37766/inplasy2020.6.0087).

### 2.2 Ethics and dissemination plans

Given that there will be no patients recruited and no data gathered from patients, ethical approval is not necessary for our research. We will publish the results of this network meta-analysis in the form of journal papers or conference papers.

### 2.3 Eligibility criteria

The PICOS principles will be consulted to establish the inclusion and exclusion criteria of this systematic review.

#### 2.3.1 Types of participants

Participants with ACI should match the diagnostic criteria of cerebral infarction revised from the 4th National Conference on Cerebrovascular Diseases in 1995, the Chinese Guidelines for the Diagnosis and Treatment of Acute Ischemic Stroke 2014 and 2018 editions or the Guidelines for the Early Management of Patients with Acute Ischemic Stroke 2019 edition.^[22–25]^

#### 2.3.2 Types of interventions and comparators

Thrombolytic, antiplatelet, anticoagulation and nutritional nerve therapy and control of blood pressure, blood glucose, and blood lipids will be considered as routine treatments. Consequently, the treatment group will consist of individuals given a type of CHI, while the control group will consist of those given routine treatment or another type of CHI. The studies that include rehabilitation, hyperbaric oxygen chamber and other physical therapies or other oral Chinese patent medicines, Chinese medicine decoctions or acupuncture, moxibustion and other TCM therapies will be excluded.

#### 2.3.3 Types of outcomes

The primary outcomes should include the National Institutes of Health Stroke Scale score, the number of effective patients (the objective criteria for cerebral infarction revised from the 4th National Conference on Cerebrovascular Diseases), and the number of adverse reactions. Secondary outcomes will include the Activity of Daily Living Scale, Barthel Index or the Water Swallow Test score.

#### 2.3.4 Types of studies

The included studies will be RCTs in this systematic review regardless of publication status and language. Animal trials, clinical experience, case reports and studies with incorrect designs or incomplete data will be excluded.

### 2.4 Data sources and retrieval strategy

Studies will be obtained from the China National Knowledge Infrastructure, Wan Fang Data, Chinese Scientific Journals Database, PubMed, Embase and Cochrane Library, regardless of publication date or language.

The databases will be retrieved by combining the subject words with random words. Taking PubMed as an example, the retrieval strategy is shown in Table 1.

**Table 1:**
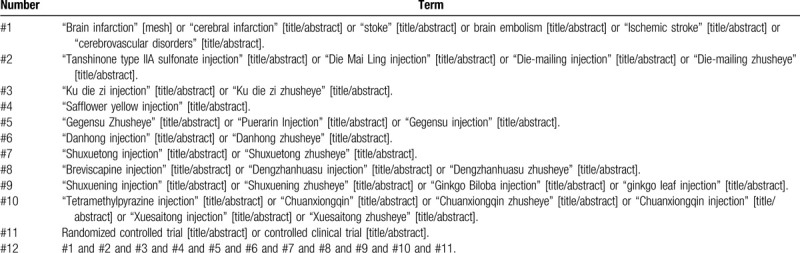
Retrieval strategy of PubMed.

The search terms will be adapted appropriately to conform to the different syntax rules of the different databases.

### 2.5 Study selection and data extraction

**Figure 1:**
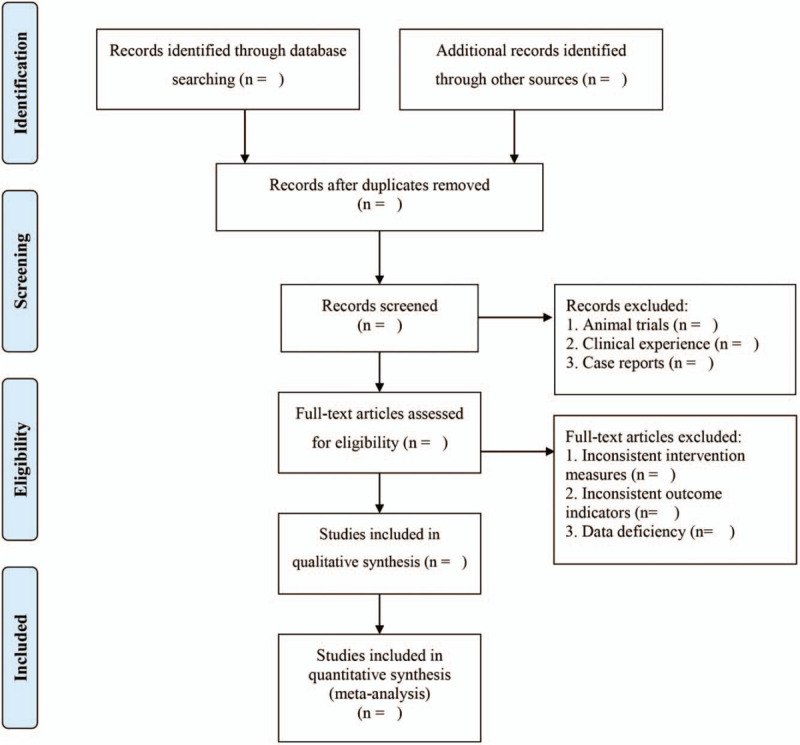
PRISMA flow chart.

EndNote X9 will be used to manage the retrieved studies. As shown in Figure 1, the study selection will be divided into 2 steps and completed by 2 researchers (BL and HS). Preliminary screening: eliminate repeated and unqualified studies by reading the title and abstract. Rescreening: read through the full text and select the studies according to the inclusion and exclusion criteria.

According to the Cochrane Handbook for Systematic Reviews of Interventions, the 2 researchers (XL and XG) will extract the author, publication time, participant number, age, race, lesion location, intervention measures, course of treatment and outcome indicators, fill in the data extraction table, and compare the results with each other.

### 2.6 Risk of bias assessment

Two researchers (BL and XG) will be designated to assess the quality of the included RCTs independently by utilizing the Cochrane Risk of Bias assessment tool. As specified by Cochrane Handbook V.5.1.0, the following sources of bias will be considered: random sequence generation, allocation concealment, participant blinding, outcome assessor blinding, incomplete outcome data, selective reporting, and other sources of bias. Each domain will be rated as having a high, low, or unclear risk of bias as appropriate.^[26]^ The 2 reviewers will resolve any disagreements through discussion, and a third reviewer (RL) may be involved if no consensus is reached.

### 2.7 Statistical analysis

#### 2.7.1 Traditional meta-analysis

Direct comparisons of CHI efficacy will be performed using Review Manager 5.3. The outcomes will be mainly represented by the mean difference or odds ratio with 95% confidence intervals, and a *P* value < .05 will be considered significant. The Cochrane Q test and I^2^ statistics were used to assess heterogeneity. When *P* < .1 or I^2^ > 50%, which indicates statistical heterogeneity, a random-effects model will be used to calculate the outcomes; otherwise, a fixed-effects model will be considered.

#### 2.7.2 Network meta-analysis

A network evidence diagram will be drawn to visually represent the comparisons between the studies. The size of the nodes represents the number of participants, and the thickness of the edges represents the number of comparisons. Stata 14.2 and WinBUGS 1.4.3 Software will be used to carry out Bayesian network meta-analysis. Bayesian inference will carried out using the Markov chain Monte Carlo method, the posterior probability will be inferred from the prior probability, and estimation and inference will be assumed when Markov Chain Monte Carlo reaches a stable convergence state. As a result, the rank of the CHI effect will be presented by the surface under the cumulative ranking curve.

Inconsistencies between direct and indirect comparisons will be evaluated using the node splitting method.^[21]^ The choices between fixed- and random-effect models and between consistent and inconsistent models will be made by comparing the deviance information criteria for each model.^[27,28]^

#### 2.7.3 Subgroup and sensitivity analysis

If there is high heterogeneity in the included studies, we will perform subgroup analyses to explore the differences in age, sex, race, lesion location, and course of the disease/treatment.

To ensure robustness of the combined results, sensitivity analyses will be performed to assess the impact of studies with a high risk of bias. We will compare the results to determine whether lower-quality studies should be excluded.

#### 2.7.4 Publication biases.

We will use funnel plots to identify whether there will be small study bias if 10 or more studies are included. Asymmetry in the funnel plot will suggest the possibility of small study effects, and the results of analysis will be explained cautiously.

### 2.8 Quality of evidence

The grading of recommendations, assessment, development, and evaluation approach will be used in evaluating evidence quality. Considerations of evidence quality assessment include study limitation, consistency of effect, imprecision, indirectness, and publication bias. The evidence quality will be classified into 4 levels (high, medium, low, and very low).^[29]^

## 3 Discussion

Although CHIs have been widely used in the treatment of ACI as a substitution therapy, due to the lack of evidence-based medical research, the clinical selection of CHIs still relies on the subjective experience of physicians.

Through the rigorous design of the experimental scheme and objective evaluation of the level of evidence, our study will provide a more reliable basis for future clinical decisions and guidance in developing CHI-based therapies.

## Author contributions

**Conceptualization:** Runmin Li, Ying Li, Yuanxiang Liu and Jiguo Yang.

**Data curation:** Bingchen Li, Haiyang Sun, Xinyu Liu, Xin Ge.

**Formal analysis:** Runmin Li and Ying Li.

**Methodology:** Yuanxiang Liu and Jiguo Yang.

**Software:** Bingchen Li and Haiyang Sun.

**Supervision:** Yuanxiang Liu and Jiguo Yang.

**Writing – original draft:** Runmin Li and Ying Li.

**Writing – review & editing:** Yuanxiang Liu and Jiguo Yang.
